# Suppression of senescent metabolism of adipose tissue by rebalancing mitochondrial homeostasis via a selective drug delivery system

**DOI:** 10.1186/s12951-026-04594-w

**Published:** 2026-06-02

**Authors:** Liwei Wang, Kaicheng Xu, Ziye Guo, Xiaoyong Wu, Di Wang, Liang Chen, Donghua Huang, Kaile Wu, Yubin Zhao, Minjun Yao, Liming Zheng, Xiaobo Yang, Chenyi Ye, Wushi Cui, Wanli Li, An Liu, Mao Zhang, Jianbin Xu

**Affiliations:** 1 Department of Orthopedic Surgery, the Second Affiliated Hospital, Zhejiang University School of Medicine, Hangzhou, 310009 P. R. China; 2 Key Laboratory of Motor System Disease Research and Precision Therapy of Zhejiang Province, Hangzhou, 310009 P. R. China; 3 Orthopedics Research Institute of Zhejiang University, Hangzhou, 310009 P. R. China; 4 Department of Emergency Medicine, Second Affiliated Hospital of Zhejiang University School of Medicine, Hangzhou, 310009 China; 5 Key Laboratory of The Diagnosis and Treatment of Severe Trauma and Burns of Zhejiang Province, Hangzhou, 310009 China; 6 Clinical Research Center for Emergency and Critical Care Medicine of Zhejiang Province, Hangzhou, 310009 China

**Keywords:** Adipose tissue, Extracellular vesicles, Senescence-associated secretory phenotype, Motor performance

## Abstract

**Background:**

Aging is characterized by a progressive decline in physiological function. Among various organs, adipose tissues play an important regulator of systemic metabolism and energy homeostasis. Age-associated alterations in adipose tissue are closely linked to organismal aging. Targeting senescent adipose tissue has therefore emerged as a potential strategy for mitigating age-related dysfunction. The purpose of this study was to develop and evaluate a selective drug delivery system for anti-aging therapy.

**Results:**

We report a targeted anti-ageing platform based on extracellular vesicles functionalized with the P3 peptide, enabling efficient delivery of dasatinib and quercetin to aged adipose tissue. This platform effectively inhibits senescence-associated secretory phenotype (SASP) in both aged adipocytes and adipose explants in vitro, concomitant with restoration of specific adipocyte function. Furthermore, systemic administration in aged mice contributes to improved physical performance and basal metabolic capacity, alongside attenuation of adipose tissue senescence. Mechanistically, these effects are probably mediated by drug-induced activation of mitophagy and the consequent reprogramming of cellular energy metabolism.

**Conclusion:**

These findings underscore the pivotal role of adipose tissue in systemic aging processes. The developed delivery platform effectively targets senescent adipose tissue, suppresses SASP, restores physiological function, and enhances organismal physical performance. This work proposes a conceptual framework linking aging and metabolism, offering a promising strategy for organ- or tissue-specific anti-aging interventions.

**Graphical Abstract:**

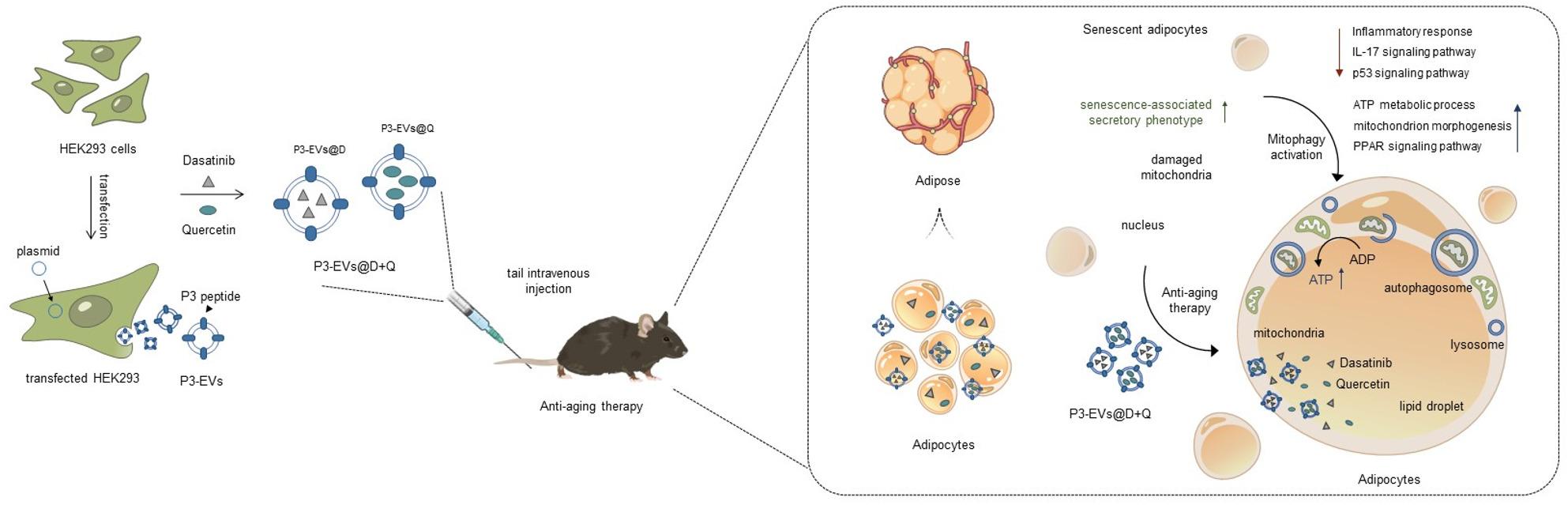

**Supplementary Information:**

The online version contains supplementary material available at 10.1186/s12951-026-04594-w.

## Background

Aging is an inevitable biological process characterized by the progressive decline of physiological functions and is recognized as a major risk factor for numerous human diseases [1, 2]. Among its most evident manifestations is the deterioration of physical performance, including reduced muscle strength, endurance, and mobility [3], which profoundly diminishes the quality of life in older individuals [4, 5]. As a systemic disorder [6], aging affects multiple organs or tissues through the disruption of cellular homeostasis and repair mechanisms [7, 8]. For instance, aging is commonly associated with impaired cardiac function [9], diminished hepatic detoxification capacity [10], as well as loss of skeletal muscle mass [11]. However, despite being a widely distributed and metabolically active organ [12, 13], adipose tissue has often been overlooked in studies of systemic aging.

Adipose tissue serves as a central regulator of systemic metabolism and energy homeostasis [14]. It is distributed across multiple depots such as visceral, subcutaneous, and bone marrow regions [15], and consists primarily of brown and white adipose tissue [16], which function in energy expenditure and storage, respectively [17]. Recent researches have further highlighted the crucial endocrine functions of adipose tissue, underscoring its role as a metabolic hub that actively influences physiological and pathological processes during aging [17, 18]. With advancing age, adipose tissue undergoes extensive remodeling characterized by increases in both cell number and size, enhanced fibrosis, mitochondrial dysfunction, and alterations in the adipokine secretion profile [19, 20]. Moreover, the senescence-associated secretory phenotype (SASP), a complex mixture of proinflammatory cytokines, chemokines, and proteases released by senescent adipocytes [21, 22], is markedly elevated during aging [18]. The resulting chronic inflammation originating from aged adipose tissue progressively disseminates throughout the body, accelerating functional decline across various organs and ultimately leading to systemic deterioration [23]. Therefore, it is essential to develop strategies for intervening in senescent adipose tissue to achieve effective systemic anti-aging therapies.

Currently, multiple anti-aging therapies have been reported. Small molecule drugs, such as rapamycin and metformin, are commonly applied for delaying senescence [24, 25]. Various senolytic strategies employing combinatorial drug treatment were proposed to intervene senescent cells in adipose tissue [26, 27]. Additionally, cell-based therapies like stem cell treatment, have been employed to promote the repair of aged tissues [28], and CAR-T cells have been utilized to selectively eliminate specific senescent cell populations [29]. However, most of these anti-aging interventions rely on systemic administration, lacking tissue specificity and thereby posing challenges such as off-target effects [24, 30], poor bioavailability, and potential adverse reactions [31]. Thus, to address these challenges, it is imperative to design a drug delivery system with sustained stability, high biocompatibility, and efficient specificity for adipose tissue targeting.

**Fig. 1:**
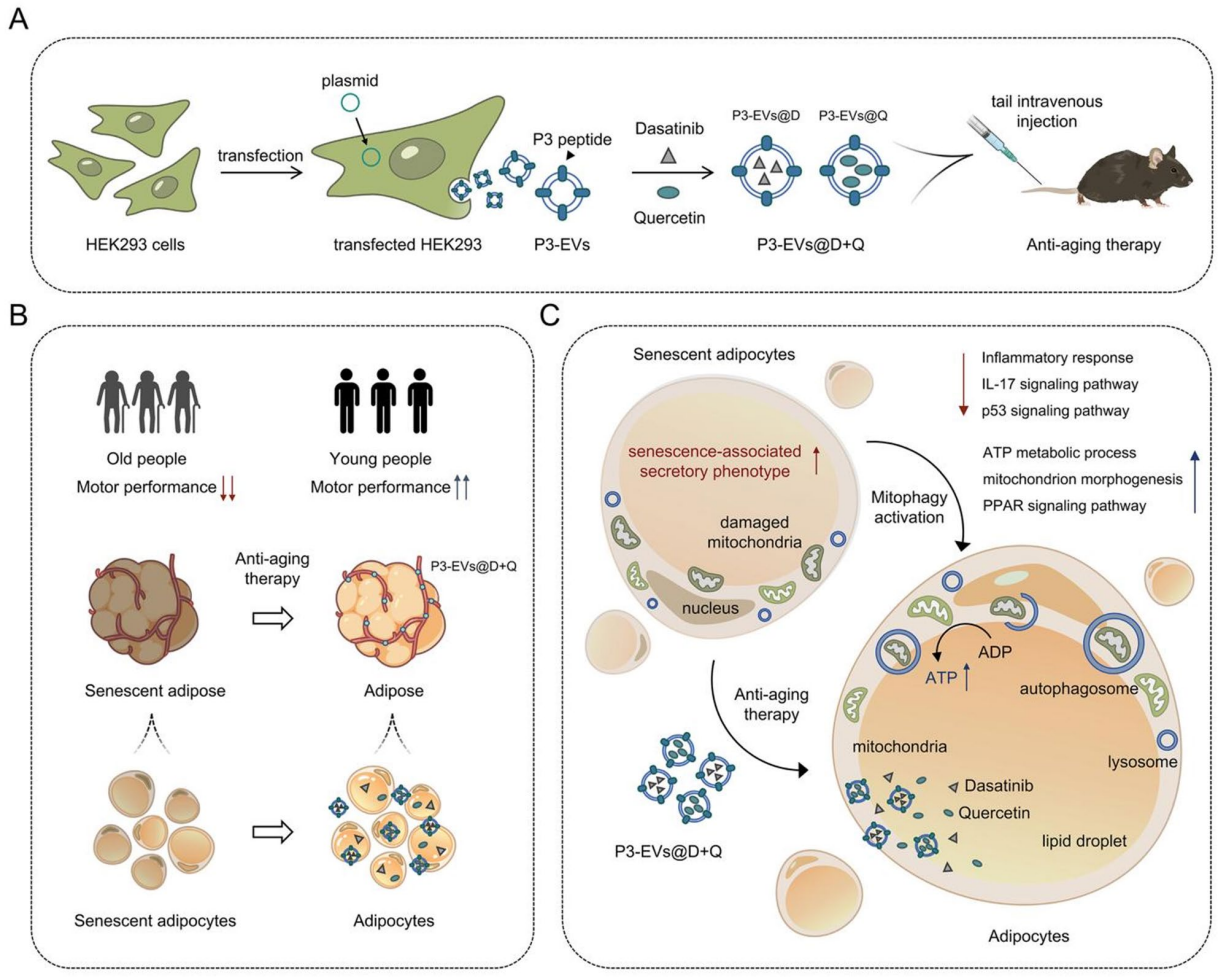
Schematic illustration.(**A**) Preparation and construction of functionalized extracellular vesicles P3-EVs@D + Q. (**B**) P3-EVs@D + Q selectively targets senescent adipose tissue and adipocytes, attenuates senescence-associated phenotypes, and consequently enhances organismal motor performance. (**C**) P3-EVs@D + Q reprograms mitochondrial energy metabolism in senescent adipocytes probably through activation of mitophagy, which in turn suppresses deleterious signaling pathways and promotes the maintenance of adipocyte-specific functions

In this study, we established a drug delivery strategy derived from extracellular vesicles (EVs) functionalized with the P3 targeting peptide, endowing the system with selective tropism for adipose tissue. Dasatinib and quercetin were encapsulated into EVs to achieve sustained release and exert anti-senescent effects (Fig. 1A). Our findings demonstrated that this engineered system effectively suppresses SASP in senescent adipose tissue both in vitro and in vivo, thereby enhancing the adipocyte function. Moreover, this intervention was also found to remarkably improve the motor performance in elderly mice (Fig. 1B). Mechanistically, these benefits may arise from drug-induced activation of mitophagy, leading to the rebalance of energy homeostasis in senescent adipocytes (Fig. 1C). Overall, our study aimed to selectively attenuate senescence phenotypes of aged adipose tissue, and these findings provided a promising platform for organ/tissue-specific anti-aging therapy on the basis of senescence-metabolism interaction.

## Materials and methods

### Plasmid transfection and retroviral infection

The sequence encoding the P3 peptide (CKGGRAKDC) was directly fused to the pDisplay vector via a flexible peptide linker (GGGGS) 3 (signal peptide, Igκ chain leader sequence; HA, hemagglutinin epitope tag (sequence YPYDVPDYA); Myc, Myc epitope (sequence EKKLISEEDL); PDGFR transmembrane domain, derived from the platelet-derived growth factor receptor). The pDisplay vector was purchased from Invitrogen. The plasmid was subsequently transfected into HEK293 cells (SNL015, SUNNCELL, China) via Lipofectamine 3000(L3000075, Invitrogen, USA) according to the manufacturer’s instructions.

### Animals and in vivo treatment

Male C57BL/6J mice (18 months old) were purchased from the Shanghai Slack Experimental Animal Center. The mice were housed in a controlled environment with a 12-hour light/dark cycle (lights started at 7:00) and had unrestricted access to food and water. All experiments were approved by the Animal Ethics and Welfare Committee of the Second Affiliated Hospital, Zhejiang University School of Medicine. The approved animal ethics and welfare number is (no. 2022 − 146). All animal care and handling procedures were conducted in accordance with the Guide for the Care and Use of Laboratory Animals by the National Institutes of Health.

C57BL/6J mice were randomly divided into 4 groups (*n* = 6) and treated with phosphate-buffered saline (PBS), free dasatinib plus quercetin (D + Q), EV-loaded dasatinib plus quercetin (EVs@D + Q), or P3-EVs loaded with dasatinib plus quercetin (P3-EVs@D + Q) once a week for 2 months. For each mouse, the oral dosage of dasatinib was 5 mg/kg, and the oral bioavailability of dasatinib was 14%. Therefore, the intravenous dosage of dasatinib for mice was 0.7 mg/kg. The reference dose of quercetin for human oral treatment is 14.29 mg/kg, with a human oral bioavailability of 2%. The dose of quercetin for human intravenous injection is 0.286 mg/kg. According to the pharmacological scaling formula, the mouse dose is equal to 9.1 times the human dose. Therefore, the intravenous dosage of quercetin for mice was 2.6 mg/kg.

### Isolation and culture of stromal vascular fraction (SVF) cells

The SVF was isolated from mouse inguinal adipose tissue as previously described [32], with minor modifications. Briefly, mice were euthanized under pentobarbital anesthesia, and the skin of the lower limbs was incised to expose the white adipose tissue located bilaterally in the inguinal region. The adipose tissue was carefully lifted using forceps and separated from the surrounding skin with surgical scissors. The harvested fat pads were transferred into pre-cooled PBS and gently washed with a pipette to remove attached hair and blood residues. The tissue was then minced into small pieces and digested in collagenase type A (Roche, USA) at 37 °C with gentle agitation for 45–60 min. Following digestion, the suspension was filtered and centrifuged at 800 × g for 5 min to pellet the SVF cells.

The cell pellet was resuspended and washed once with Dulbecco’s Modified Eagle Medium (DMEM), followed by another centrifugation step (800 × g, 5 min) to remove residual enzymes and debris. The final SVF pellet was resuspended in complete DMEM (supplemented with 10% fetal bovine serum, 100 U/mL penicillin, and 100 µg/mL streptomycin) and seeded into T25 culture flasks. The medium was replaced every 48 h thereafter.

To induce adipogenic differentiation of SVF cells, the culture medium was replaced with Medium A when the cells reached approximately 90% confluence. Cells were incubated in Medium A for 2 days, followed by a switch to Medium B for an additional 4 days. Medium A consisted of the basal culture medium supplemented with 1 µM dexamethasone (HY-14648, MCE, USA), 0.5 mM IBMX (HY-12318, MCE), 2 µM rosiglitazone (Hy-17386, MCE), and 10 µg/mL insulin (HY-P0035, MCE), while Medium B contained 10 µg/mL insulin as the only supplement. This induction maintenance cycle was repeated until the appearance of abundant intracellular lipid droplets was observed under microscopy. Adipocyte differentiation was confirmed by Oil Red O staining (Beyotime, China), according to the manufacturer’s instructions.

### Oxidative stress–induced senescence and SA-β-Gal staining

To induce cellular senescence, SVF-derived adipocytes were exposed to 100 µM hydrogen peroxide (H₂O₂; diluted in growth medium) for 24 h. After treatment, senescence-associated β-galactosidase (SA-β-gal) staining was performed using a commercial kit (Beyotime) according to the manufacturer’s instructions.

Briefly, the culture medium was removed, and cells were washed once with PBS. Cells were then fixed with 1 mL of β-galactosidase fixative solution for 15 min at room temperature. After fixation, the cells were washed three times with PBS, and 1 mL of freshly prepared SA-β-gal staining working solution was added to each well. The cells were incubated overnight at 37 °C in a CO₂-free environment. Finally, SA-β-gal-positive cells exhibiting blue staining were observed and imaged under a light microscope.

### Isolation of P3-EVs

P3-EVs were isolated from the conditioned medium of HEK293T cells transfected with the P3 peptide by differential ultracentrifugation. Briefly, culture supernatants were sequentially centrifuged at 300 × g for 10 min to remove cells, 1200 × g for 10 min to eliminate cell debris, and 10,000 × g for 30 min to remove larger vesicles and apoptotic bodies. The clarified supernatants were then ultracentrifuged at 100,000 × g for 70 min at 4 °C (Beckman Coulter Optima, USA). The resulting EV pellets were washed once with sterile PBS and collected by a second ultracentrifugation at 100,000 × g for 70 min. Finally, EVs were resuspended in PBS.

The total protein concentration of isolated P3-EVs was determined using a Mini BCA protein assay kit (Beyotime). The morphology of P3-EVs was observed using transmission electron microscopy (TEM), while their hydrodynamic size distribution was analyzed by dynamic light scattering (DLS). The surface charge (zeta potential) of the P3-EVs was also measured to assess their physicochemical properties. Furthermore, the presence of characteristic EV marker proteins, including CD9, CD63, CD81, and TSG101, was verified by Western blotting.

### Western blotting

Protein expression was analyzed by western blotting. Cells and EVs were lysed in RIPA buffer (Affinibody LifeScience, China) containing protease inhibitors (Thermo Fisher Scientific, USA). Adipose tissue explants were homogenized on ice in radioimmunoprecipitation assay (RIPA) buffer supplemented with a protease inhibitor cocktail. Homogenates were centrifuged at 12,000 × g for 15 min at 4 °C, and the resulting supernatants containing total proteins were collected for subsequent analyses.

Protein concentrations were measured using a BCA assay (Beyotime). Equal amounts of protein were resolved by SDS–PAGE and transferred to PVDF membranes (Merck Millipore, USA). After blocking with 5% skim milk, membranes were incubated with primary antibodies and HRP-conjugated secondary antibodies. Signals were detected using an enhanced chemiluminescence system (Yeasen, China). The antibodies used in this study include Calnexin (1:1000, A15631, ABclonal, China), CD63 (1:1000, A19023, ABclonal), CD9 (1:2000, A1703, ABclonal), CD81 (1:2000, A22983, ABclonal), TSG101 (1:1000, A2216, ABclonal), HA (1:10000, #2367, Cell Signaling Technology), p16 (1:1000, ab51243, Abcam, UK), p21 (1:1000, ab188224, Abcam), p62 (1:10000, ab109012, Abcam), LC3B (1:2000, 81004-1-RR, Proteintech, USA), and TOM20 (1:5000, 11802-1-AP, Proteintech).

### In vivo fluorescence imaging of P3-EVs distribution

EVs and P3-EVs were labeled using a DiO fluorescent dye (Beyotime). Briefly, EVs suspended in PBS were incubated with 5 µM DiO dye for 30 min, followed by centrifugation at 12,000 × g for 70 min to remove unbound dye. The resulting EV pellets were resuspended in PBS and intravenously injected into C57BL/6J mice (*n* = 3). At 24 h post-injection, mice were anesthetized and perfused with 0.9% cold saline via cardiac perfusion to eliminate circulating EVs. Heart, liver, spleen, lungs, kidneys and adipose tissue (including inguinal, epididymal, and subcutaneous fat) were subsequently harvested, and fluorescence signals were detected using an in vivo imaging system (IVIS).

### Loading dasatinib or quercetin into P3-EVs

Dasatinib or quercetin was separately loaded into P3-EVs using an ultrasound-based method. Briefly, mixtures of dasatinib with P3-EVs or quercetin with P3-EVs were subjected to sonication using a 505-type Sonic Dismembrator. The sonication parameters were set at 20% amplitude for six cycles, with each cycle consisting of 30s on and 30s off, for a total of 3 min. A 2 min cooling interval was applied between consecutive cycles to prevent overheating. Following sonication, the mixtures were incubated at 37 °C for 30 min to facilitate resealing of the exosomal membranes. Free (unloaded) dasatinib or quercetin was removed by ultracentrifugation at 100,000 × g for 70 min, which was repeated twice to ensure purity. Finally, drug-loaded P3-EVs were quantified by measuring the intrinsic fluorescence of dasatinib at 324 nm or quercetin at 374 nm using a fluorescence spectrophotometer.

### In vitro drug release kinetics

To determine the drug release profile of D and Q from the P3-EVs, a known concentration of P3-EVs suspension was placed in a dialysis bag (in triplicate), immersed in PBS and incubated in a 37 °C shaking water bath at 60 rpm. The amount of D/Q released from P3-EVs was estimated by recording the absorbance of the supernatant at 324/374 nm.

### Biocompatibility evaluation of P3-EVs@D + Q

To evaluate the biocompatibility of P3-EVs on adipocytes, 100 µg DiO-labelled P3-EVs@D + Q were added to adipocytes differentiated from SVF cells and incubated at 37 °C for 24 h. The cells were then washed thoroughly with PBS to remove unbound P3-EVs@D + Q, followed by nuclei staining using a hoechst dye (Beyotime). The cellular uptake of EVs was visualized using a confocal laser scanning microscope (CLSM; Olympus FV1000). Additionally, to assess the potential cytotoxic effects of P3-EVs@D + Q, live/dead viability staining (Beyotime) was conducted in accordance with the manufacturer’s protocol following co-incubation of the cells with unlabeled P3-EVs@D + Q. The results were also observed by a CLSM.

### Adipose tissue explants culture

Adipose tissue was excised from the epididymal fat pads of 18-month-old male C57BL/6 mice. The tissue was cut into small pieces and washed three times with PBS. The adipose tissue was then cultured in media containing sodium pyruvate (1 mM), glutamine (2 mM), MEM vitamins, MEM nonessential amino acids, and antibiotic supplements supplemented with P3-EVs@D (1 µM) and P3-EVs@Q (20 µM). After 48 h, the adipose explants were washed and prepared for further investigations.

### Real-time PCR

Total RNA was extracted from samples using RNAiso reagent (Takara, Kusatsu, Japan) following the manufacturer’s instructions. The purified RNA was reverse transcribed into complementary DNA (cDNA) using the Double-Strand cDNA Synthesis Kit (Takara). Quantitative real-time PCR (qRT-PCR) was then performed with SYBR Green PCR Master Mix (Takara) on an Applied Biosystems™ 7500 system (Applied Biosystems, Waltham, MA, USA). The primer sequences used are shown in Supplementary Table S1. Relative gene expression was calculated via the ΔΔCT method, where the fold difference was calculated via the expression 2^‒ΔΔCt method.

### Behavioral assessment

To evaluate locomotor activity and gait performance, mice underwent a series of motor ability tests, the open-field test and gait analysis. All measurements were performed at least 3 days after the final treatment.

The maximum walking speed of the mice was assessed using an accelerating RotaRod system (TSE Systems, Chesterfield, MO). On days 1, 2, and 3, the mice were trained at constant speeds of 4, 6, and 8 rpm, respectively, for 200 s. On the test day, the rotation speed started at 4 rpm and gradually increased to 40 rpm over 5 min. The speed at which each mouse fell off the RotaRod was recorded. Data were averaged from three trials per mouse and normalized to the baseline speed.

For the hanging endurance test, mice were placed on a 2-mm thick metal wire suspended 35 cm above a padded surface, with only their forelimbs allowed to grasp the wire. Hanging performance was expressed as hanging time (seconds) × body weight (grams), and the average of three trials was calculated for each mouse.

Endurance exercise capacity was assessed by a treadmill running test. Prior to the experiment, mice were acclimated to the treadmill for 2 days, running at 5 m/min for 3 min and then at 7 m/min for 2 min. On the test day, mice ran on a treadmill set at a fixed 5° incline, beginning at 5 m/min for 2 min, after which the speed was increased by 2 m/min every 2 min until exhaustion. Exhaustion was defined as the inability to resume running despite mild electrical and mechanical stimuli. Running distance was recorded, and total work (kJ) was calculated according to the following formula: mass (kg) × g (9.8 m/s²) × distance (m) × sin (5°).

The open-field test was used to evaluate locomotor activity and exploratory behavior. Each mouse was individually placed in a square open-field arena (40 × 40 cm, opaque walls) for 10 min of free exploration. Locomotor trajectories were recorded with a video-tracking system. All experiments were conducted under uniform lighting and noise conditions, and testing was performed at the same time of day to minimize circadian influences.

Gait analysis was conducted using a DigiGait Imaging System (Mouse Specifics Inc, USA). Mice were trained to walk voluntarily on an illuminated transparent treadmill belt while a high-speed camera positioned underneath recorded paw placements. Each mouse was tested until it could complete at least three continuous walks. Parameters of the left hind of each mouse was recorded, including average ground contact pressure, foot support time footprint distance and step rate.

### Indirect calorimetry

Indirect calorimetry was performed in 18-month-old C57BL/6 mice (*n* = 3) treated with P3-EVs@D + Q. Mice were approximately weight-matched across experimental groups. Prior to data acquisition, animals were acclimatized to PhenoMaster metabolic cages (TSE Systems) without running wheels for 3 days. Gas exchange parameters, including oxygen consumption (VO₂) and carbon dioxide production (VCO₂), as well as food and water intake and locomotor activity (beam breaks), were recorded at 20-minute intervals over a 5-day period. Data from the first 24 h were excluded to allow for acclimation to the metabolic cages. Measurements from the subsequent 4 days were averaged and resampled into hourly intervals to generate a representative 24-hour profile.

Data points deemed physiologically implausible, likely due to system leaks or sensor malfunction, were excluded from analysis. The daily amplitude of respiratory exchange ratio (RER), energy expenditure, and locomotor activity was defined as the difference between the maximum and minimum hourly values within a 24-hour period, averaged across the 4-day recording window. Due to higher variability in feeding behavior, the daily amplitude of energy intake was calculated by dividing the 24-hour period into four 6-hour intervals and determining the difference between the maximum and minimum interval values.

### ELISA

ELISA kits were used according to the manufacturer’s instructions. Briefly, standards and samples were added to antibody-precoated wells, covered with a sealing film, and gently shaken for 10 s, followed by incubation at 37 °C for 90 min. After washing twice, HRP-conjugated streptavidin was added and incubated at 37 °C for 30 min, followed by three washes. TMB substrate solution was then added and incubated at 37 °C for 10–20 min. Finally, 50 µL of stop solution was added, and the absorbance was immediately measured at 450 nm. Sample concentrations were calculated from the standard curve.

### Cytokine chip analysis

Visceral adipose tissue, spleen, muscle, heart, and lung were collected from experimental mice immediately after euthanasia. Tissues were rinsed briefly in cold phosphate-buffered saline (PBS) to remove residual blood and then minced into small pieces on ice. The tissue samples were homogenized in appropriate volumes of cold lysis buffer compatible with the Bio-Plex assay and centrifuged at 12,000 × g for 10–15 min at 4 °C to obtain the clarified supernatants.

Cytokine levels in the tissue homogenates were measured using the Bio-Plex Pro Mouse Chemokine Panel 31-Plex (Bio-Rad, USA) following the manufacturer’s instructions. Briefly, magnetic beads conjugated with capture antibodies for 31 chemokines were added to a 96-well filter plate. Tissue supernatants, standards, and quality controls were added, and the plate was incubated at room temperature with shaking for 30 min. After washing to remove unbound proteins, biotinylated detection antibodies were added for 30 min, followed by incubation with streptavidin–phycoerythrin (SA-PE) for 10 min. Beads were washed, resuspended in assay buffer, and analyzed using a Bio-Plex 200 system (Bio-Rad, USA). Cytokine concentrations were calculated based on standard curves generated from the supplied standards.

### Histological analysis

Tissue samples were harvested from experimental mice, fixed in 4% paraformaldehyde at 4 °C overnight, dehydrated through graded ethanol, cleared in xylene, and embedded in paraffin. Paraffin-embedded tissues were sectioned at 4–5 μm thickness and mounted onto glass slides. To confirm the absence of adverse effects resulting from P3-EVs@D + Q in vivo, slides of heart, lung, liver, kidney, and spleen of mice were prepared for HE staining.

For immunohistochemistry analysis, slides of each adipose tissue underwent processing with primary antibodies against p16 (1:100, ab51243, Abcam), p21 (1:1000, ab188224, Abcam), p53 (1:100, ab131442, Abcam), H2A (1:1000, ab11174, Abcam), followed by incubation of the corresponding HRP-conjugated secondary antibodies. All these slides were subjected to observation and imaging using a light microscopy (Aperio GT 450, Leica, GER).

### Mitochondrial function assessment

Mitochondrial function was evaluated using several assays, including ATP content measurement, oxygen consumption rate (OCR) analysis, and assessment of mitochondrial membrane potential.

ATP content was measured using an ATP Determination Kit (Thermo Fisher Scientific, USA). Briefly, the standard reaction solution was prepared and its background luminescence was recorded. Diluted ATP standards or samples were then added, and luminescence was measured. Background values were subtracted, and ATP concentrations were calculated based on the standard curve.

Mitochondrial membrane potential was assessed using JC-1 dye (5,5’,6,6’-tetrachloro-1,1’,3,3’-tetraethylbenzimidazolylcarbocyanine iodide; Invitrogen). Briefly, cells were incubated with 2 µM JC-1 at 37 °C in the dark for 20 min. Fluorescence microscopy was then used to observe the ratio of red to green fluorescence, which reflects mitochondrial membrane potential.

Mitochondrial respiration was assessed by measuring the OCR using a Seahorse XF Analyzer (Agilent Technologies, USA) according to the manufacturer’s instructions. Briefly, cells were seeded into Seahorse XF microplates at an appropriate density and allowed to adhere overnight. Prior to measurement, the culture medium was replaced with assay medium and equilibrated for 45–60 min. Mitochondrial function was evaluated by sequential injection of modulators to determine basal respiration, ATP-linked respiration, maximal respiration, spare respiratory capacity, proton leak, and non-mitochondrial respiration.

### Fluorescence staining

For immunofluorescence staining of slides from adipose explants, primary Ki67 antibody (1:1000, 28074-1-AP, Proteintech) was first used, followed by incubation of fluorescent secondary antibody marked in green. DAPI (Thermo Fisher Scientific) was used to label the cell nucleus. The staining results were imaged by a CLSM and quantified by ImageJ software (NIH, Bethesda, MD, USA).

To observe the activated mitophagy in adipocytes after P3-EVs@D + Q treatment, cells were washed by PBS and then incubated with mitotracker (Thermo Fisher Scientific) and lysotracker (Thermo Fisher Scientific) at 37 °C for 30 min. After removal of unbound fluorescent dye by extensive washing, fluorescence signals were visualized using a CLSM and quantitative analysis of fluorescence intensity was performed with ImageJ. For co-localization analysis of lysosomes and LC3B (1:500, 81004-1-RR, Proteintech), cells were pre-labeled with lysotracker as described above, fixed with 4% paraformaldehyde, and subsequently subjected to LC3B immunofluorescence staining following standard procedures.

### Statistical analysis

Statistical analysis was performed via GraphPad Prism software, and all the data are presented as the means ± standard deviations (SDs) from at least three independent biological replicates (*n* ≥ 3). For in vitro investigations, n denotes independent experiments; for in vivo studies, n represents the number of animals per group. Comparisons between two groups were performed using unpaired two-tailed Student’s t-tests, while multiple group comparisons were analyzed by one-way or two-way ANOVA with appropriate post hoc tests, as indicated. *, *P* < 0.05; **, *P* < 0.01; ***, *P* < 0.001; ns, no significance.

## Results and discussion

### Adipose tissue plays a pivotal role in systemic aging processes

**Fig. 2:**
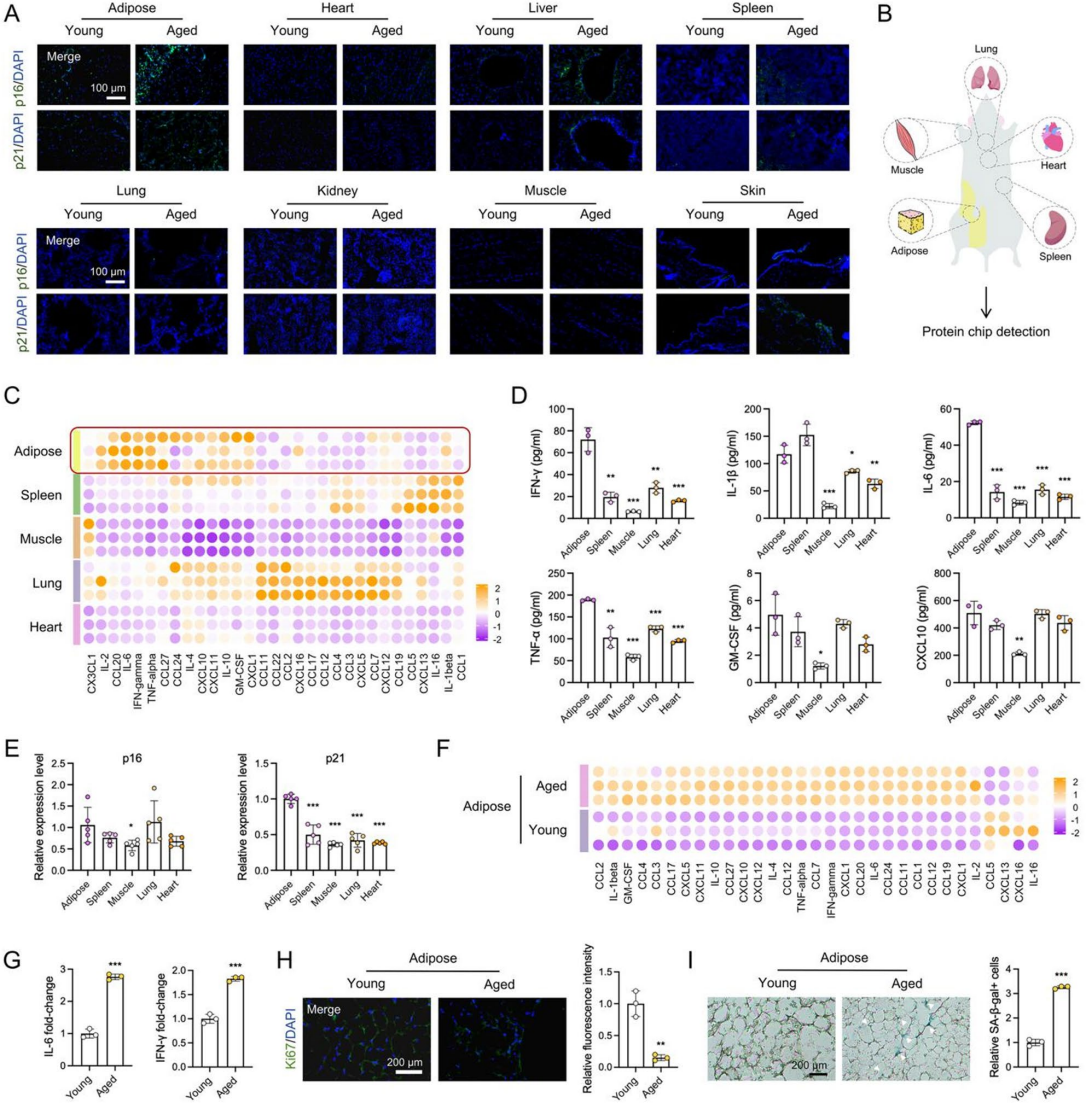
Adipose tissue plays an important role in systemic aging processes. (**A**) Representative immunofluorescence staining images of the senescence-associated markers p16 and p21 in major tissues and organs, including adipose tissues, heart, liver, spleen, lung, kidney, muscle, and skin. (**B**) Schematic representation of protein chip detection using samples from five different organs or tissues in aging mice. (**C**) Heatmap visualization of protein chip profiling across five distinct senescent organs and tissues. (**D**)Quantifications of SASP-related cytokines (including IFN-γ, IL-1β, IL-6, TNF-α, GM-CSF, and CXCL10) detected by cytokine chip analysis. (**E**) Relative mRNA expression levels of p16 and p21 in different organs or tissues. (**F**) Heatmap results of protein chip analysis between young and aged adipose tissues. (**G**) quantifications of SASP-related cytokines (IL-6 and IFN-γ) determined by cytokine chip analysis. (**H**) Representative immunofluorescence images and corresponding quantification of Ki67 in young and aged adipose tissues. (**I**) Representative SA-β-gal staining images and corresponding quantification of young and aged adipose tissues. White arrows indicate β-gal-positive cells. SASP, senescence-associated secretory phenotype. *P < 0.05; **P < 0.01; ***P < 0.001; compared to the adipose group

Aging is a systemic process that affects multiple organs and tissues [20, 33]. To systematically characterize senescence features across different organs, we performed immunofluorescence staining of two canonical senescence markers, p16 and p21 [21], in eight tissues from young (6-week-old) and aged (18-month-old) mice (Fig. 2A). Although 18-month-old mice are widely considered to model early-stage aging and may not fully capture the physiological and pathological complexity of advanced aging [34, 35], our study is specifically focused on early aging, which represents a more therapeutically tractable stage that can be mitigated or even reversed by pharmacological interventions. As shown in Fig. 2A, both p16 and p21 exhibited markedly elevated signals in adipose tissue and liver of aged mice, suggesting that these tissues may play prominent roles in systemic aging. In contrast, only sporadic signals were observed in the lung, spleen, and epidermal layer of the skin, while both markers were barely detectable in corresponding tissues from young mice.

To further investigate organ-specific differences in senescence-associated protein expression, we conducted protein chip profiling in heart, lung, muscle, adipose tissue, and spleen (Fig. 2B). Heatmap analysis revealed that, compared with other organs, senescence-associated biomarkers were most prominently altered in adipose tissue and lung, with adipose tissue showing the most pronounced changes (Fig. 2C). Notably, senescence-associated secretory phenotype (SASP) factors were markedly upregulated in aged adipose tissue. SASP is a hallmark feature of cellular senescence, characterized by the secretion of various chemokines, cytokines, and growth factors [36, 37], which can reinforce senescence in an autocrine manner and propagate it to neighboring cells via paracrine signaling [38]. Moreover, SASP has been implicated in promoting age-associated inflammation and tumorigenesis [39–41], thereby playing a critical role in the progression of systemic aging.

Quantitative analysis showed in Fig. 2D further demonstrated that key inflammatory cytokines, including interleukin 6 (IL-6), interferon γ (IFN-γ), and tumor necrosis factor α (TNF-α), reached approximately 60, 90, and 200 pg/mL, respectively, in adipose tissue, substantially higher than in other organs. In addition, other SASP-related factors, such as interleukin 1β (IL-1β), granulocyte-macrophage colony-stimulating factor (GM-CSF), and CXC chemokine ligand 10 (CXCL10), were also significantly enriched in aged adipose tissue, indicating robust activation of the SASP program (Fig. 2D). Consistently, mRNA expression analysis showed that both p16 and p21 were significantly upregulated in adipose tissue compared with other organs (Fig. 2E), further confirming its pronounced senescent phenotype.

To exclude the possibility that adipose tissue intrinsically exhibits a higher basal SASP level in young mice, we directly compared protein profiles between young and aged adipose tissues (Fig. 2F). The results revealed clear and widespread upregulation of SASP-associated factors upon aging. For example, IL-6 levels increased by approximately 2.7-fold and IFN-γ by nearly 2-fold in aged adipose tissue (Fig. 2G). Functionally, immunofluorescence staining of Ki67 demonstrated a marked reduction in proliferative capacity in aged adipose tissue, as evidenced by decreased fluorescence intensity (Fig. 2H). Conversely, the number of SA-β-gal-positive cells was significantly increased (Fig. 2I), further supporting the accumulation of senescent cells. Taken together, these findings indicate that adipose tissue exhibits a pronounced senescent phenotype characterized by excessive SASP activation and reduced regenerative capacity, suggesting it may play an indispensable role in systemic aging processes.

Adipose tissue has been widely reported to be closely associated with the aging status of other organs and tissues through the secretion of diverse bioactive factors [42]. Notably, recent studies suggest that age-related immune alterations can be detected early in white adipose depots [43, 44]. Consistent with these observations, our protein chip analysis revealed that, even at the early stage of aging, several representative SASP-related proinflammatory cytokines, particularly IL-6, TNF-α and IFN-γ, were remarkably enriched in senescent adipose tissue compared to lung (Fig. 2B-D). Moreover, the expression level of p21 was also highest in adipose tissue among the examined organs (Fig. 2E), further highlighting its pronounced senescence-associated features. Given its high vascularization, abundant blood supply [45], and relative lack of anatomical barriers to drug delivery [18], adipose tissue represents a readily accessible and pharmacologically tractable target for systemic intervention. Collectively, these findings support the rationale for selecting adipose tissue as a key target tissue in our anti-aging strategy.

### A precise drug delivery system was constructed and characterized

Considering the importance of adipose tissue in aging, development of effective anti-aging therapies targeting senescent adipose tissue becomes a promising alternative which might result in systemic body improvement owing to its wide distribution. However, few effective strategies have been proposed. Drug therapy, such as metformin and rapamycin [46], are limited by safety concerns and potential side effects, as well as poor tissue specificity [47]; dietary restriction and physical exercise are relatively safe approaches, but they typically require prolonged intervention periods and their efficacy varies considerably among individuals [47]. Therefore, it is necessary to design a composite drug delivery system towards aged adipose tissue.

**Fig. 3:**
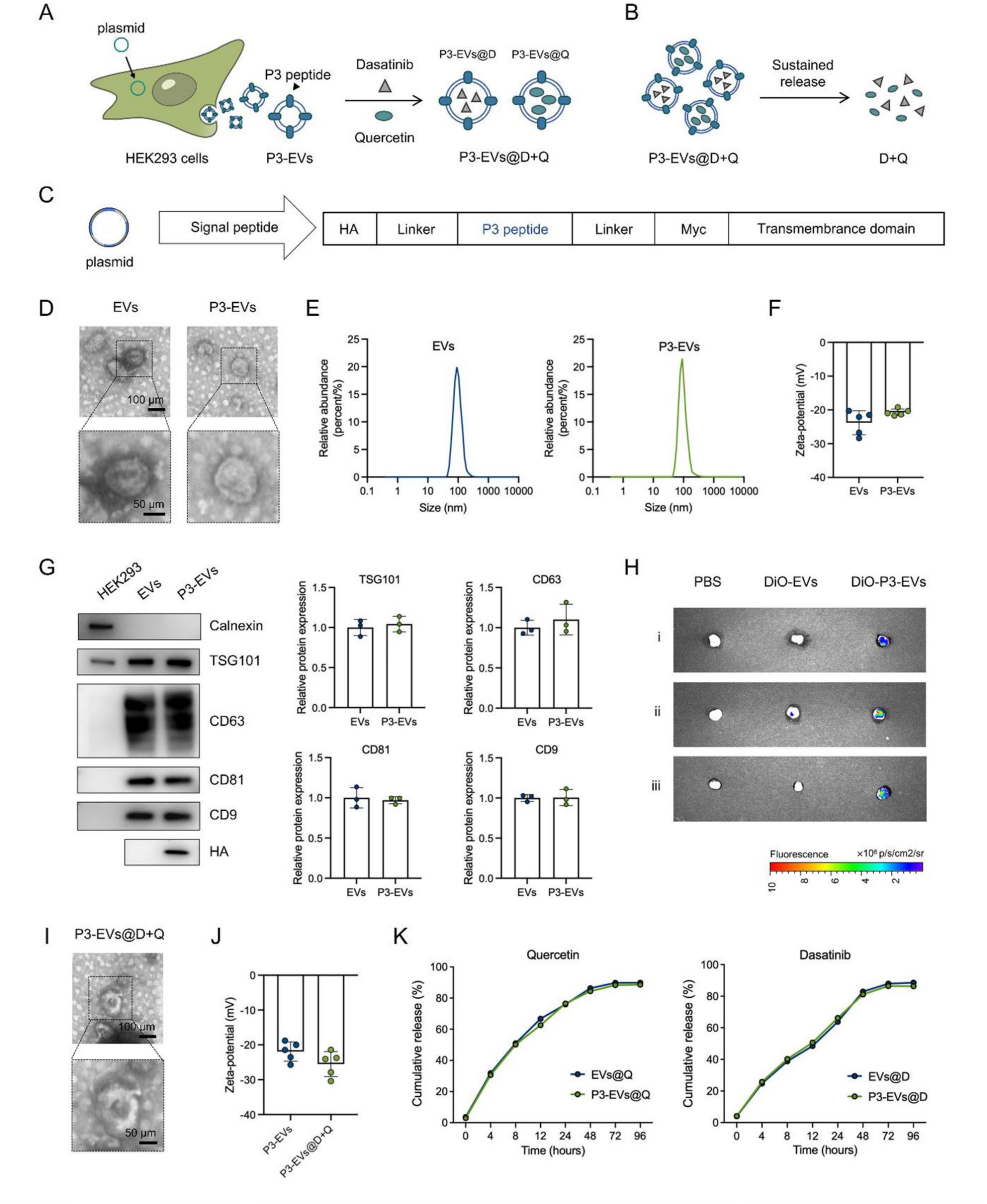
Preparation and characterization of functionalized P3-EVs@D + Q. (**A-B**) Schematic diagrams of construction of P3-EVs@D + Q and its sustained release of dasatinib and quercetin (abbreviated as D and Q, respectively). (**C**) Construction of the plasmid used for HEK293 cell transfection to obtain the EVs expressing P3 peptide. (**D**) Representative TEM images of EVs and P3-EVs. The parts marked by the dashed box are enlarged below the relevant original images. (**E**) DLS analysis of purified EVs and P3-EVs. (**F**) Surface zeta potential of the two different EVs. (**G**) Representative western blotting images and quantifications for canonical EV markers, including TSG101, CD63, CD81 and CD9. Calnexin served as a negative control marker and HEK293 cellular proteins served as the control sample. (**H**) Representative fluorescence images of inguinal adipose tissues after treatment with DiO-labelled EVs or P3-EVs via tail intravenous injection in mice (i, ii, and iii denote three independent biological replicates). (**I**) Representative TEM images of P3-EVs@D + Q. The part marked by the dashed box is enlarged below. (**J**) Surface zeta potential of P3-EVs@D + Q. (**K**) Sustained release curves of dasatinib or quercetin encapsulated in EVs or P3-EVs. EVs, extracellular vesicles

EVs have been widely employed as drug delivery carriers due to their excellent biocompatibility, low immunogenicity, and prolonged circulation time [48]. They also possess the ability to cross certain biological barriers [49]. Therefore, we selected EVs as the drug delivery platform in this study. For endowing EVs with the essential function of targeting adipose tissue, we focused on the P3 peptide [50], which specifically binds to adipose vasculature through the interaction with membrane protein prohibitin, according to the recent researches [27, 51]. A plasmid was constructed to transfect the HEK293T cell line for the generating stable P3 peptide-modified EVs (P3-EVs), which were subsequently utilized for drug delivery (Fig. 3A-B). The plasmid facilitated the expression of P3 peptide on the plasma membrane through the transmembrane domain of the platelet-derived growth factor receptor (Fig. 3C). The sequence encoding the P3 peptide was cloned and inserted into the pDisplay vector and the resulting fusion protein was expressed in HEK293T cells via transfection.

Unlike simple physical mixing or chemical conjugation modifications to attach molecules to the EVs membrane, this gene editing approach preserves the vesicle membrane integrity and ensures the stability of the targeting molecules. Notably, this design is supposed to be extended to modify EVs with short peptides or single-chain antibodies targeting any other tissues/cells, providing an immediate platform for the specific modification of EVs.

We first characterized the physicochemical properties of the modified P3-EVs. Transmission electron microscopy (TEM) images showed that both EVs and P3-EVs exhibited similar cup-shaped morphologies and intact membrane structures (Fig. 3D). Dynamic light scattering (DLS) analysis revealed that P3 peptide modification did not induce any marked changes in particle size, with mean diameters of 90.6 nm for EVs and 94.2 nm for P3-EVs (Fig. 3E). In addition, zeta potential measurements indicated no significant difference between the two groups (Fig. 3F). To confirm the identity and modification of P3-EVs, western blotting was then performed. The results demonstrated comparable expression levels of classical exosomal markers, including TSG101, CD63, CD81, and CD9 in both EVs and P3-EVs, while only P3-EVs expressed the HA-tagged protein, confirming successful conjugation of the P3 peptide (Fig. 3G). Next, we evaluated the in vivo adipose-targeting capability of P3-EVs. The results showed that DiO-labeled P3-EVs exhibited strong fluorescence signals across multiple adipose depots, including inguinal (Fig. 3H), epididymal, and subcutaneous fat (Supplementary Fig. 1). Only minimal signal of P3-EVs was detected in non-adipose organs, with a low level observed in the liver (Supplementary Fig. 2). In contrast, unmodified EVs displayed negligible accumulation in adipose tissues, with only sporadic and weak signals observed in a small number of fats, while showing pronounced accumulation in the liver, consistent with nonspecific uptake (Supplementary Fig. 1–2). These findings support that P3 peptide modification significantly enhances adipose tissue tropism of EVs in vivo, with no obvious off-targeting.

To ensure potent anti-aging efficacy of the P3-EVs-based drug delivery system, we next focused on the well-established drug combination dasatinib and quercetin (D + Q), which was firstly identified and remained the most extensively characterized senolytic regimen [25]. Dasatinib, a tyrosine kinase inhibitor, have been shown to eliminate accumulated senescent cells in vivo, whereas quercetin, a naturally occurring plant flavonoid, exerts antioxidant and anti-inflammatory effects [52–54]. The combination of these two agents was believed to act synergistically, thereby enhancing overall anti-aging efficacy [55, 56]. However, the poor water solubility of dasatinib and quercetin results in low oral bioavailability [57]. Moreover, the lack of specificity toward particular cell populations or tissues may cause systemic side effects when local therapeutic concentrations are achieved [58, 59]. Therefore, we separately loaded dasatinib and quercetin into the P3-EVs via an ultrasonic method (Fig. 3A-B) and the resulting system was named P3-EVs@D + Q. TEM and zeta potential detection were conducted to verify the membrane integrity of P3-EVs@D + Q after ultrasonic stimulation (Fig. 3I-J). No significant structural change was observed after drug loading (Fig. 3I-J). In addition, the cumulative drug release curves of dasatinib and quercetin revealed that both two drugs were gradually released from the P3-EVs with a duration of 70 h approximately (Fig. 3K), suggesting the acceptable sustained drug release kinetic of P3-EVs@D + Q.

### P3-EVs@D + Q attenuate senescence phenotypes of adipocytes in vitro

**Fig. 4:**
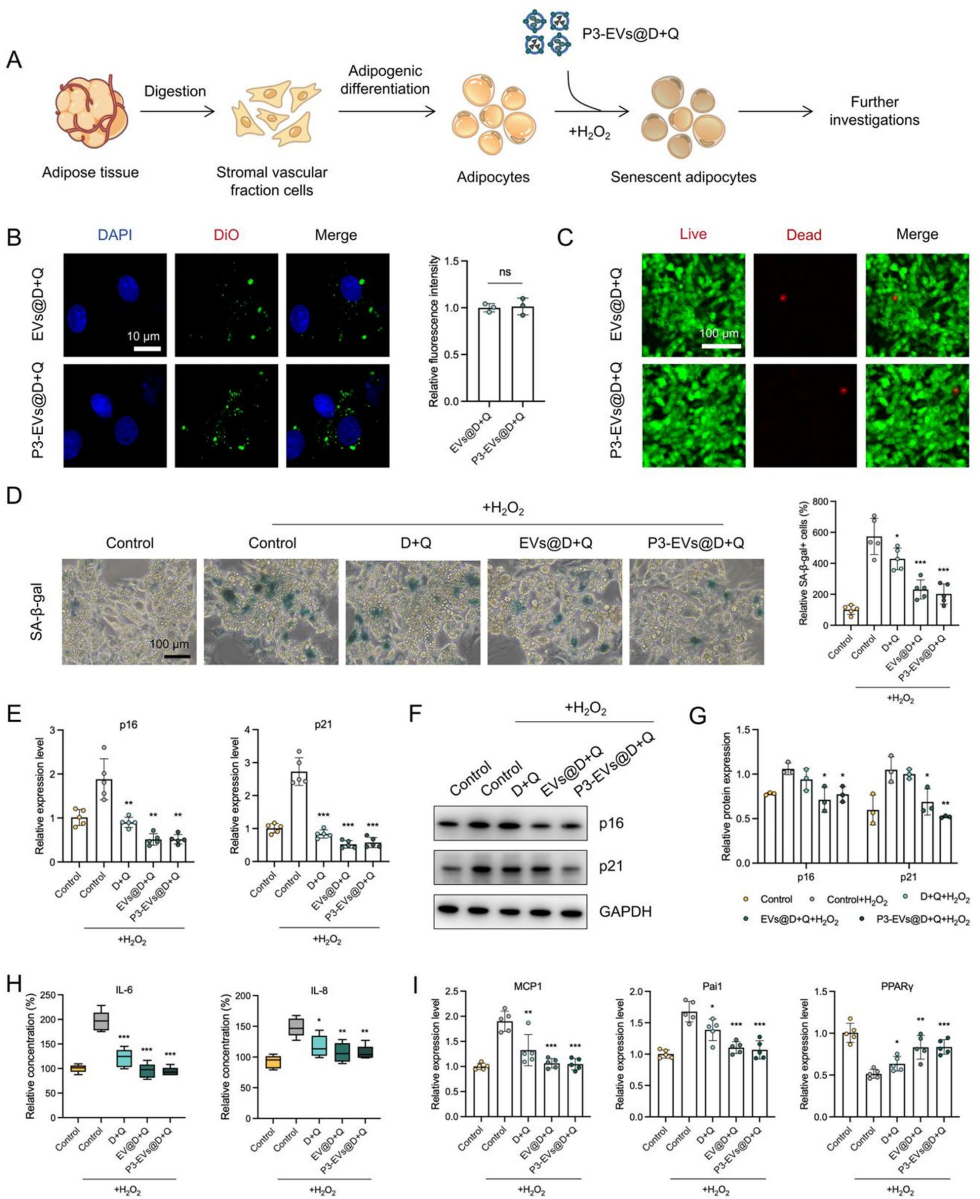
P3-EVs@D + Q attenuate senescence phenotypes of adipocytes. (**A**) Schematic diagrams of adipocyte differentiation, the P3-EVs@D + Q management and senescence induction using H_2_O_2_. (**B**) Fluorescence staining and the relevant quantification to determine the intracellular uptake of P3-EVs@D + Q. (**C**) Representative fluorescence images of live-death staining of adipocytes after P3-EVs@D + Q treatment. (**D**) The SA-β-gal staining results of senescent adipocytes with different management. (**E**) Relative mRNA expression levels of p16 and p21 in different groups. (**F**) Representative western blotting images and (**G**) the corresponding quantifications of p16 and p21 in adipocytes treated with PBS, D + Q, EVs@D + Q and P3-EVs@D + Q. (**H**) ELISAs of two SASP-related cytokines IL-6 and IL-8 (*n* = 5). (**I**) The mRNA expression levels of SASP-related markers (MCP1 and Pai1) and adipose function-related marker (PPARγ) in different groups. SASP, senescence-associated secretory phenotype; MCP1, monocyte chemoattractant protein 1; Pai1, plasminogen activator inhibitor 1; PPARγ, peroxisome proliferator-activated receptor γ. *P < 0.05; **P < 0.01; ***P < 0.001; ns, no significance; compared to the control group treated with H_2_O_2_

The anti-aging effects of P3-EVs@D + Q were first verified using differentiated adipocytes derived from SVF cells with H_2_O_2_ treatment employed to induce cellular senescence (Fig. 4A). Oil red O staining revealed the presence of abundant intracellular lipid droplets (Supplementary Fig. 3A) and the mRNA level of peroxisome proliferator-activated receptor γ (PPARγ) and CCAAT/enhancer-binding protein α (CEBPα), two classic markers of adipogenesis [60], were elevated by 1.9-fold and 2.5-fold, respectively, compared with the control group (Supplementary Fig. 3B). These results confirmed the successful adipogenic differentiation.

After confirming the intracellular uptake and the biocompatibility of P3-EVs@D + Q (Fig. 4B-C), we next investigate their potential effects on senescent adipocytes. SA-β-gal staining showed that the numbers of positive stained senescent cells were significantly decreased in both the EVs@D + Q and P3-EVs@D + Q groups compared to the control group (Fig. 4D). Simiarly, the mRNA (Fig. 4E) and protein expression levels (Fig. 4F-G) of p16 and p21 were remarkably decreased in adipocytes treated with either EVs@D + Q or P3-EVs@D + Q. These senescence-associated indicators showed more pronounced downregulation in the two EV-based drug delivery groups compared with the free D + Q group (Fig. 4E-G), demonstrating that P3-EVs@D + Q effectively attenuate the senescent phenotype of adipocytes, and that encapsulation of dasatinib and quercetin within EVs markedly enhances their anti-senescence efficacy.

Further investigations revealed that the concentrations of SASP-associated cytokines IL-6 and IL-8 were markedly reduced in the culture supernatant of adipocytes following treatment with P3-EVs@D + Q, compared with the control (Fig. 4H). The mRNA levels of monocyte chemoattractant protein 1 (MCP-1) and plasminogen activator inhibitor 1 (Pai-1), two key components of the SASP [61, 62], were also significantly downregulated (Fig. 3I), indicating that P3-EVs@D + Q treatment effectively suppressed SASP hyperactivation in senescent adipocytes. Moreover, the expression level of PPARγ was restored following drug treatment (Fig. 4I), suggesting functional recovery of adipocytes. Notably, EVs@D + Q and P3-EVs@D + Q displayed comparable efficacy in both attenuating senescence-associated phenotypes and restoring adipocyte function. Quantitative analyses revealed no significant differences between the two groups, which likely reflects the limited functional contribution of the P3 peptide under in vitro conditions, where EVs directly access adipocytes and thus bypass the requirement for targeting-mediated tissue delivery. Collectively, these findings demonstrated that P3-EVs@D + Q were efficiently internalized by adipocytes and potently mitigated senescence phenotypes, thereby alleviating adipocyte aging and preserving cellular function.

### P3-EVs@D + Q alleviate senescence in adipose tissue explants

**Fig. 5:**
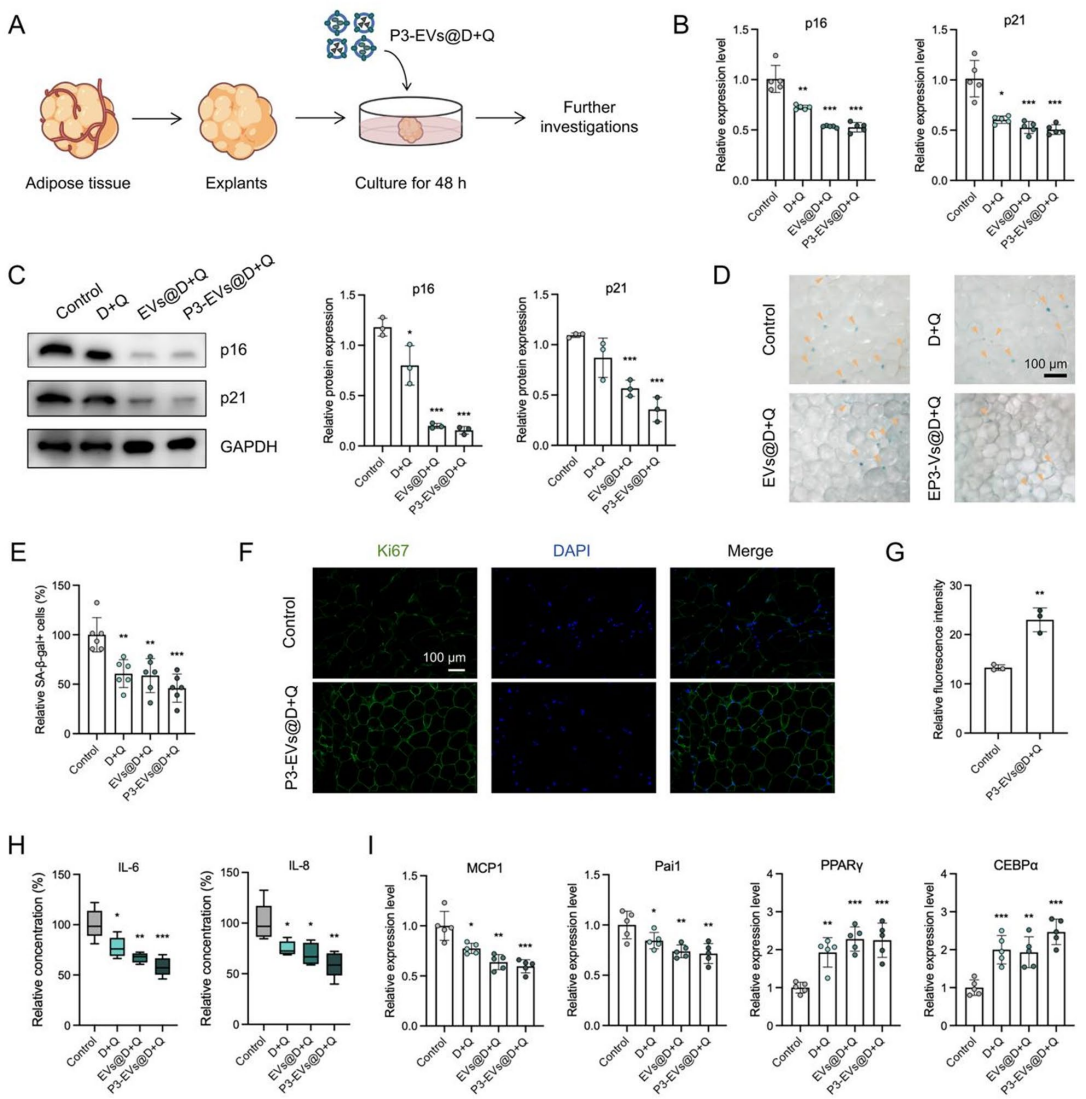
P3-EVs@D + Q suppressed senescent phenotypes of adipose tissue explants. (**A**) Schematic diagrams of adipose explants culture in vitro. (**B**) Relative mRNA expression levels of p16 and p21. (**C**) Representative western blotting images and corresponding quantifications of p16 and p21 in different groups. (**D**) Representative SA-β-gal staining images, and (**E**) corresponding quantification of adipose explants. (**F**) Representative fluorescence images and (**G**) quantitative results of Ki67 in adipose explants treated with P3-EVs@D + Q. (**H**) ELISA results of IL-6 and IL-8 (*n* = 5). (**I**) Relative mRNA expression levels of SASP-related markers (MCP1 and Pai1) and adipose function-related marker (PPARγ and CEBPα). MCP1, monocyte chemoattractant protein 1; Pai1, plasminogen activator inhibitor 1; PPARγ, peroxisome proliferator-activated receptor γ; CEBPα, CCAAT/enhancer-binding protein α. *P < 0.05; **P < 0.01; ***P < 0.001; compared to the control group

Given the complex microenvironment of adipose tissue in vivo, verifying the anti-aging effects of P3-EVs@D + Q solely through cell-based experiments was considered insufficient. Therefore, we further conducted investigations using adipose tissue explants isolated from aged mice to examine the inhibitory effects of P3-EVs@D + Q on senescence-associated metabolism (Fig. 5A).

As shown in Fig. 5B-C, both the mRNA and protein levels of p16 and p21 were significantly decreased in these two treatment groups, compared with the control group. In addition, SA-β-gal staining results showed that the number of blue-stained senescent cells in the P3-EVs@D + Q-treated group was reduced by nearly 50% relative to the control group (Fig. 5D-E), suggesting a pronounced inhibitory effect of P3-EVs@D + Q on adipose tissue senescence. Furthermore, Ki67 immunofluorescence staining demonstrated markedly enhanced green fluorescence in explants treated with P3-EVs@D + Q (Fig. 5F), fluorescence intensity approximately 1.7-fold higher than that of the control (Fig. 5G), suggesting that P3-EVs@D + Q effectively improved the proliferative capacity of senescent adipose tissue. Similarly, the concentrations of IL-6 and IL-8 (Fig. 5H), along with the mRNA expression levels of MCP1 and Pai1 (Fig. 5I), were significantly reduced across all treated groups relative to the control. In contrast, the expression levels of PPARγ and CEBPα were upregulated following P3-EVs@D + Q treatment (Fig. 5I), indicating functional improvement of adipose tissue. Consistent with the in vitro results, no significant difference was observed between EVs@D + Q and P3-EVs@D + Q in adipose tissue explants. This is likely due to the absence of systemic delivery barriers in the ex vivo setting, where EVs directly access the tissue, thus diminishing the requirement for P3-mediated targeting. Taken together, these findings demonstrated that P3-EVs@D + Q efficiently mitigate the senescent phenotype of adipose tissue explants and promote restoration of adipose function.

Previous studies have reported the anti-aging effects of D + Q across multiple organs and tissues. For instance, D + Q has been shown to alleviate aging-associated bone metabolic dysfunction [63] and to attenuate pulmonary fibrosis by eliminating senescent cells and suppressing their pro-fibrotic activity [52]. Consistently, we also observed distinct anti-aging effects of free dasatinib and quercetin on senescent adipocytes as well as adipose tissue explants. As shown in Fig. 5D-I and B-I, direct administration of these two drugs (D + Q group) moderately reduced the expression of senescent markers (p16 and p21) as well as the SASP factors. However, its inhibitory effect was limited and significantly weaker than that observed in the EVs@D + Q and P3-EVs@D + Q groups. This improvement in therapeutic efficacy is likely attributable to the reduced toxicity of free dasatinib and quercetin when encapsulated [64], and the enhanced drug stability and cellular uptake conferred by the EV and P3-EV delivery systems.

### P3-EVs@D + Q improves the motor performance of aged mice

**Fig. 6:**
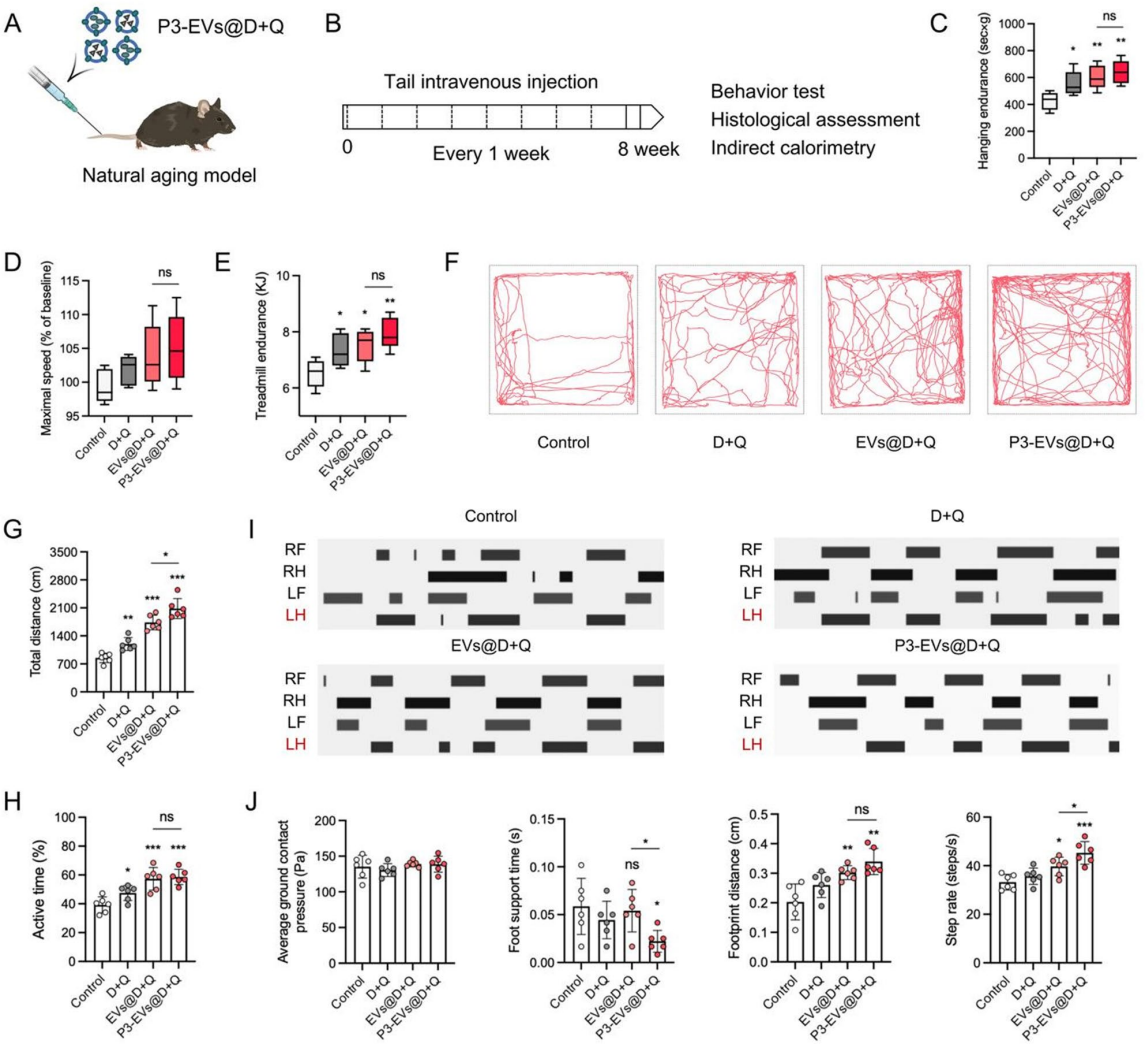
P3-EVs@D + Q improves the motor performance of aged mice. (**A-B**) Schematic diagrams of animal experiments (*n* = 6 per group). Naturally aged mice were administered with P3-EVs@D + Q via tail intravenous injection once weekly for 8 weeks, followed by behavioral tests, histological assessments, and Indirect calorimetry. (**C**) Hanging endurance, (**D**) maximal speed in movement and (**E**) treadmill endurance of aged mice. (**F**) Representative images of movement trajectory map of aged mice determined by open field test and (**G-H**) the relevant quantifications of total distance and active time percent. (**I**) Representative images of gait analysis and (**J**) the relevant quantifications of different parameters, including average ground contact pressure, foot support time footprint distance and step rate. RF, right front; RH, right hind; LF, left front; LH, left hind. *P < 0.05; **P < 0.01; ***P < 0.001; ns, no significance; compared to the control group

To evaluate the in vivo anti-aging efficacy of P3-EVs@D + Q, naturally aging mice were administered with P3-EVs@D + Q via tail vein injection once a week and behavioral tests together with histological assessments were performed at week 8 (Fig. 6A-B). Given the limited evidence regarding the systemic benefits of combating adipose tissue aging, particularly the improvements in physical performance, we specifically investigated whether alleviating adipose senescence could enhance motor function in aged mice.

As shown in Fig. 6C-E, the mice treated with EVs@D + Q and P3-EVs@D + Q exhibited modest increases in hanging endurance and treadmill endurance, whereas maximal running speed remained unchanged. Moreover, the mice administered with P3-EVs@D + Q presented enhanced locomotor activity and exercise capacity in open field test. As shown in Fig. 6F, the mice treated with P3-EVs@D + Q displayed denser and more disorganized movement trajectories, whereas the control mice showed fewer and sparser tracks. Consistently, corresponding quantifications revealed that the total movement distance (Fig. 6G) and active time percent (Fig. 6H) in the P3-EVs@D + Q group were significantly increased compared to those in the control group. Further gait analysis showed that the foot support time of the left hind limb of mice in P3-EVs@D + Q group was notably reduced and the footprint distance and step rate were markedly increased in the contrary, compared to those in the control (Fig. 6I-J). However, the average ground contact pressure presented no significant difference between the four groups (Fig. 6J). Taken together, these findings indicated that administration of P3-EVs@D + Q effectively alleviated the aging phenotype in aged mice, contributing to improved motor performance and a more youthful exercise capacity.

Notably, several behavioral indicators, including hanging endurance, maximal running speed, treadmill endurance, active time in open field test and footprint distance in gait analysis, were comparable in the P3-EVs@D + Q group to those observed in the EVs@D + Q and even the D + Q groups (Fig. 6B-J). This similarity could be attributed to several factors. Firstly, 18-month-old mice used in this study are generally considered to be at the early stage of aging [65], and their motor decline may not yet be fully apparent, leading to a limited and easily saturated effect of drug treatment on motor performance. Secondly, certain motor parameters, such as paw pressure, show minimal differences between young and aged mice [66], resulting in no significant change after P3-EVs@D + Q treatment. On the other hand, the therapeutic effect may also be related to the sample size of aged mice and treatment duration, as a 8-week intervention might not be sufficient for cellular improvements to translate into observable behavioral outcomes. Collectively, these factors may potentially lead to the behavioral results observed among the treatment groups in this study.

### P3-EVs@D + Q enhances energy metabolism in aged mice

Considering that adipose tissue is critically linked to energy balance, indirect calorimetry analysis was then performed towards aged mice between the control and the P3-EVs@D + Q group. After treatment for 8 weeks, respiratory exchange ratio (RER) of aged mice showed no significant difference between groups across the diurnal cycle (Supplementary Fig. 4A), indicating that substrate utilization remained largely unchanged. In contrast, oxygen consumption (VO₂) was significantly increased in treated mice (Supplementary Fig. 4B and H), accompanied by a corresponding elevation in energy expenditure (EE, Supplementary Fig. 4C), suggesting enhanced whole-body metabolic activity upon P3-EVs@D + Q treatment. Analysis of locomotor activity revealed no significant difference in X-axis movement counts (Supplementary Fig. 4D) or velocity between the two groups (Supplementary Fig. 4F). However, total distance traveled was significantly increased in P3-EVs@D + Q-treated mice (Supplementary Fig. 4E), indicating improved locomotor capacity rather than changes in movement pattern or speed. Additionally, body weight of mice in the two different groups remained comparable during the measurement period (Supplementary Fig. 4G and I). This result excluded the differences in body mass as a confounding factor for energy expenditure. Collectively, these findings indicate that P3-EVs@D + Q treatment enhances energy expenditure in aged mice, and this metabolic shift is accompanied by improved locomotor performance.

### P3-EVs@D + Q alleviates the senescent phenotype of adipose tissue in vivo

**Fig. 7:**
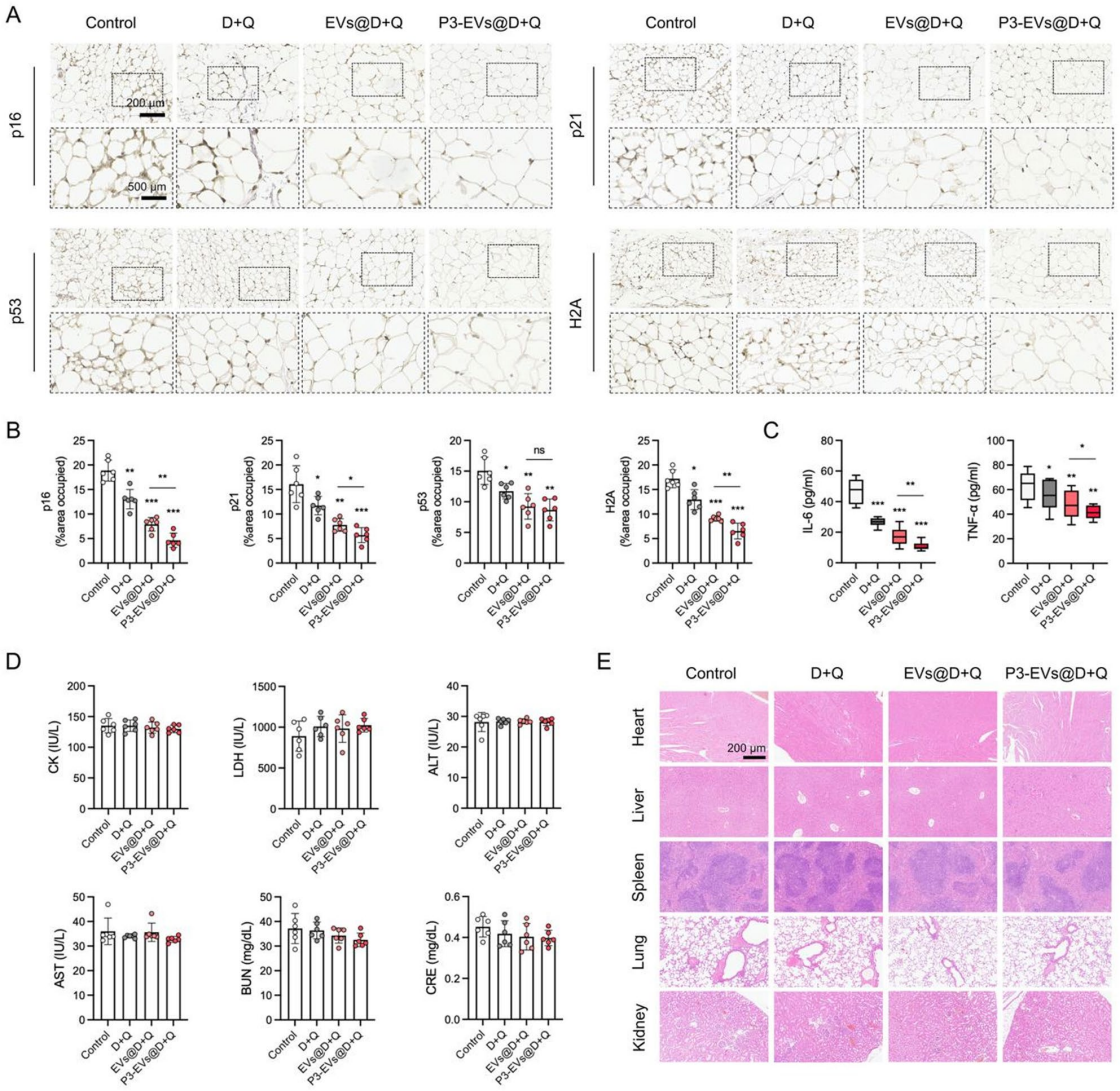
Administration of P3-EVs@D + Q attenuates the senescent phenotype of adipose tissue in aged mice. (**A**) Representative images of immunohistochemical staining and (**B**) the corresponding quantifications of senescence related markers, including p16, p21, p53 and H2A. The regions outlined by the black dashed boxes are enlarged below in (**A**). (**C**) ELISAs of two SASP-related cytokines IL-6 and IL-8 (*n* = 6 per group). (**D**) Assessment of cardiac, liver and kidney functions of aged mice after P3-EVs@D + Q administration using CK, LDH, ALT, AST, BUN and CRE levels. (**E**) Histological evaluation of major organs, including heart, liver, spleen, lung and kidney in different groups by HE staining. CK, creatine kinase; LDH, lactate dehydrogenase; ALT, alanine aminotransferase; AST, aspartate aminotransferase; BUN, blood urea nitrogen; CRE, creatinine. *P < 0.05; **P < 0.01; ***P < 0.001; ns, no significance; compared to the control group

Immunohistochemical staining of senescence-related markers, including p16, p21, p53 and H2A, was next conducted to evaluate the in vivo anti-aging effects of P3-EVs@D + Q (Fig. 7A). A larger number of positive cells stained by the four markers were identified in the control group, suggesting the aging state of adipose tissue in aged mice (Fig. 7A), consistent with the above protein chip results in Fig. 4. In contrast, the positive cell numbers were significantly reduced after P3-EVs@D + Q administration with only a few scattered brown-stained cells observed across all four indicators (Fig. 7A). Relevant quantifications further verified this trend (Fig. 7B), suggesting that P3-EVs@D + Q efficiently alleviated the senescent phenotype of adipose tissue in aged mice. Moreover, concentrations of SASP components including IL-6 and IL-8 in adipose tissue determined by ELISAs also displayed a remarkable reduce in P3-EVs@D + Q treated mice, compared to the control (Fig. 7C). Especially, the IL-6 concentration of the P3-EVs@D + Q group decreased by 76.9% relative to that of the control group (Fig. 7C). Of note, compared with the EVs@D + Q group, the P3-EVs@D + Q group exhibited stronger anti-aging effects across multiple indicators (Fig. 7A-C), strongly suggesting that P3-EVs, unlike conventional EVs, were able to efficiently target senescent adipose tissue to promote local drug accumulation and thereby enhance anti-aging efficacy.

To assess the biosecurity of the drug delivery system in vivo, serum biochemistry analysis were performed and the results indicated that no remarkable negative effect was imposed on cardiac, liver and kidney after P3-EVs@D + Q administration (Fig. 7D). Moreover, no obvious pathological change was observed in HE staining results of heart, liver, spleen, lung, or kidney (Fig. 7E), demonstrating that the excellent biocompatibility of P3-EVs@D + Q. Altogether, these findings verified that administration of P3-EVs@D + Q via tail vein injection effectively inhibited the senescent state of adipose tissue of aged mice without obvious side effect on other organs.

### P3-EVs@D + Q exert anti-aging effects by enhancing mitochondrial energy metabolism

**Fig. 8:**
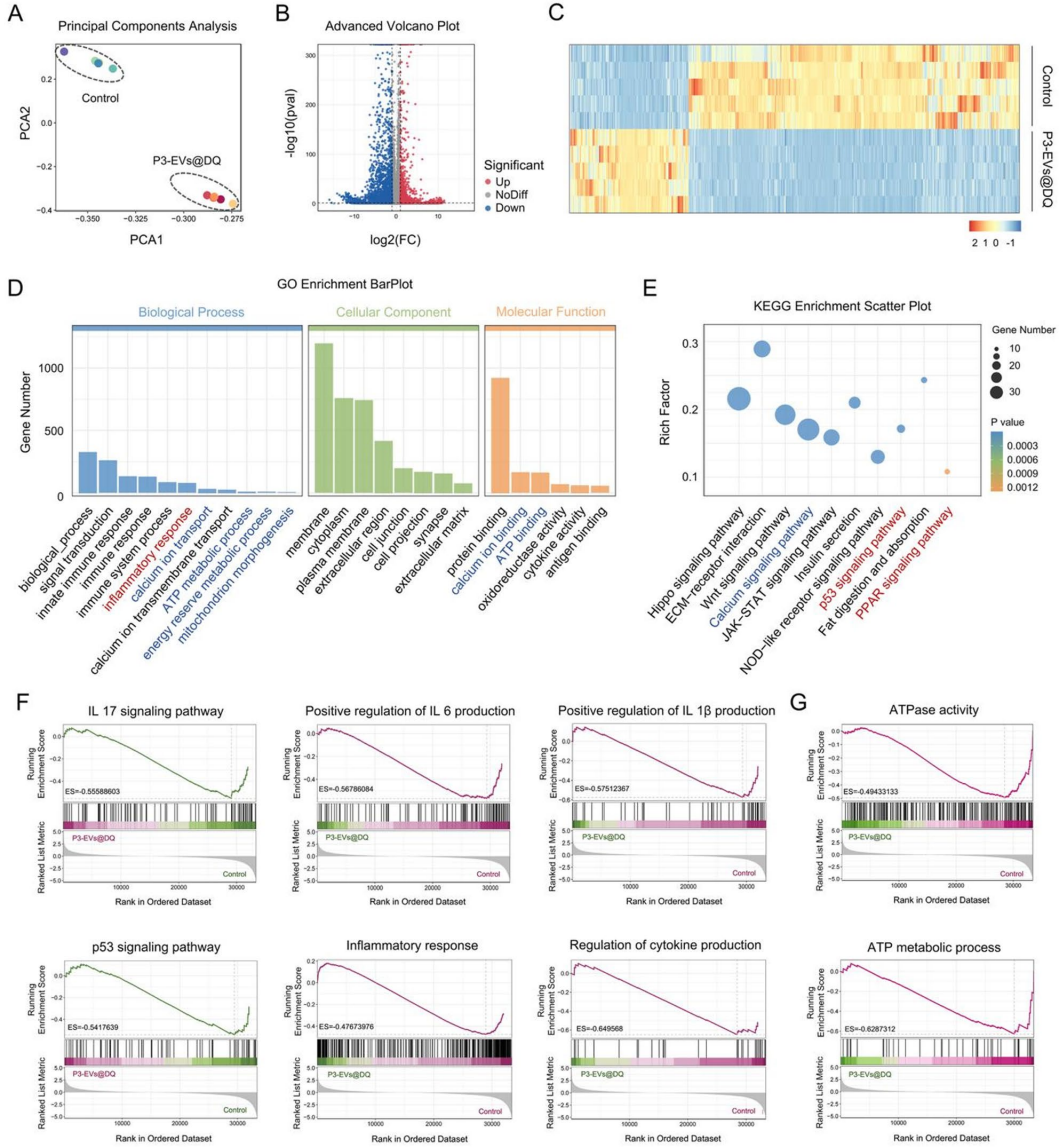
Transcriptomics results of senescent adipose tissue after P3-EVs@D + Q treatment. (**A**) Principal components analysis of the P3-EVs@D + Q and control groups. (**B**) Volcano plot of differential gene expression between the two groups. (**C**) Heatmap of differential gene expression in the P3-EVs@D + Q and control groups. (**D**) GO and (**E**) KEGG enrichment analysis in the two different groups. (**F**) GSEA of SASP-related genes. (**G**) GASE of genes related to energy metabolism

To figure out the potential mechanism of the anti-aging effects induced by our drug delivery system, adipose tissues in the control and P3-EVs@D + Q groups were then collected and processed for transcriptome analysis. The results of Principal component analysis suggested acceptable specimen stability in the two groups (Fig. 8A). Pronounced changes in transcript levels were observed, as well as in the levels of differentially expressed genes (DEGs) (Fig. 8B-C). Approximately 3300 DEGs were identified based on the filtering criteria (Fig. 8C). Gene Ontology (GO) annotation analysis revealed that DEGs were enriched in known biological processes involved in inflammatory response (Fig. 8D), which is regarded closely associated with SASP [8]. Additionally, Kyoto Encyclopedia of Genes and Genomes (KEGG) enrichment analysis indicated that most DEGs were enriched in p53 signaling pathway and PPAR signaling pathway (Fig. 8E), related to senescent phenotype and adipose functions [8]. Gene set enrichment analysis (GSEA) provided further verifications. As shown in Fig. 8F, notable enrichment of genes involved in pathways such as IL-17 signaling, p53 signaling, inflammatory response, positive regulation of IL-6 and IL-1β production and regulation of cytokine production was identified. These findings demonstrated remarkable changes in the senescent phenotype of adipose tissue from aged mice following P3-EVs@D + Q treatment, highly consistent with the above in vivo investigation results (Fig. 7).

Furthermore, we discovered a significant enrichment of signaling pathways related to mitochondrial energy metabolism. For example, GO analysis revealed that DEGs were enriched in several biological processes including calcium ion transport, ATP metabolic process, energy reserve metabolic process and mitochondrion morphogenesis, as well as molecular functions including calcium ion binding and ATP binding (Fig. 8D). In addition, differential enrichment of DEGs were also observed in gene sets such as ATPase activity and ATP metabolic process, according to GSEA (Fig. 8G). These results led us to hypothesize that P3-EVs@D + Q primarily exert their effects by modulating energy metabolism.

Actually, P3-EVs@D + Q precisely improves adipose tissue aging mainly through the stable, sustained release of D + Q. As previously reported, dasatinib is closely involved in mitochondria-mediated apoptotic pathways [67] and has been shown to promote mitochondrial autophagy in keratinocytes by modulating the PI3K/Akt/mTOR signaling axis [68]. Additionally, quercetin has been reported to probably activate the AMPK/SIRT1/PGC1α pathway to restore mitochondrial biogenesis in neuronal cells [69], and also to facilitate the clearance of damaged mitochondria via regulating the PINK1/Parkin-dependent mitophagy in renal tubular epithelial cells [70]. However, the potential mechanisms by which the two drugs, especially quercetin, influence mitochondrial metabolism in senescent adipocytes are not yet fully understood. Based on previous researches and our GO analysis, which identified the enrichment of mitochondrion morphogenesis (Fig. 8D), regarded as a key process in mitophagy [71], we consequently proposed that P3-EVs@D + Q probably enhanced energy metabolism by activating mitophagy.

**Fig. 9:**
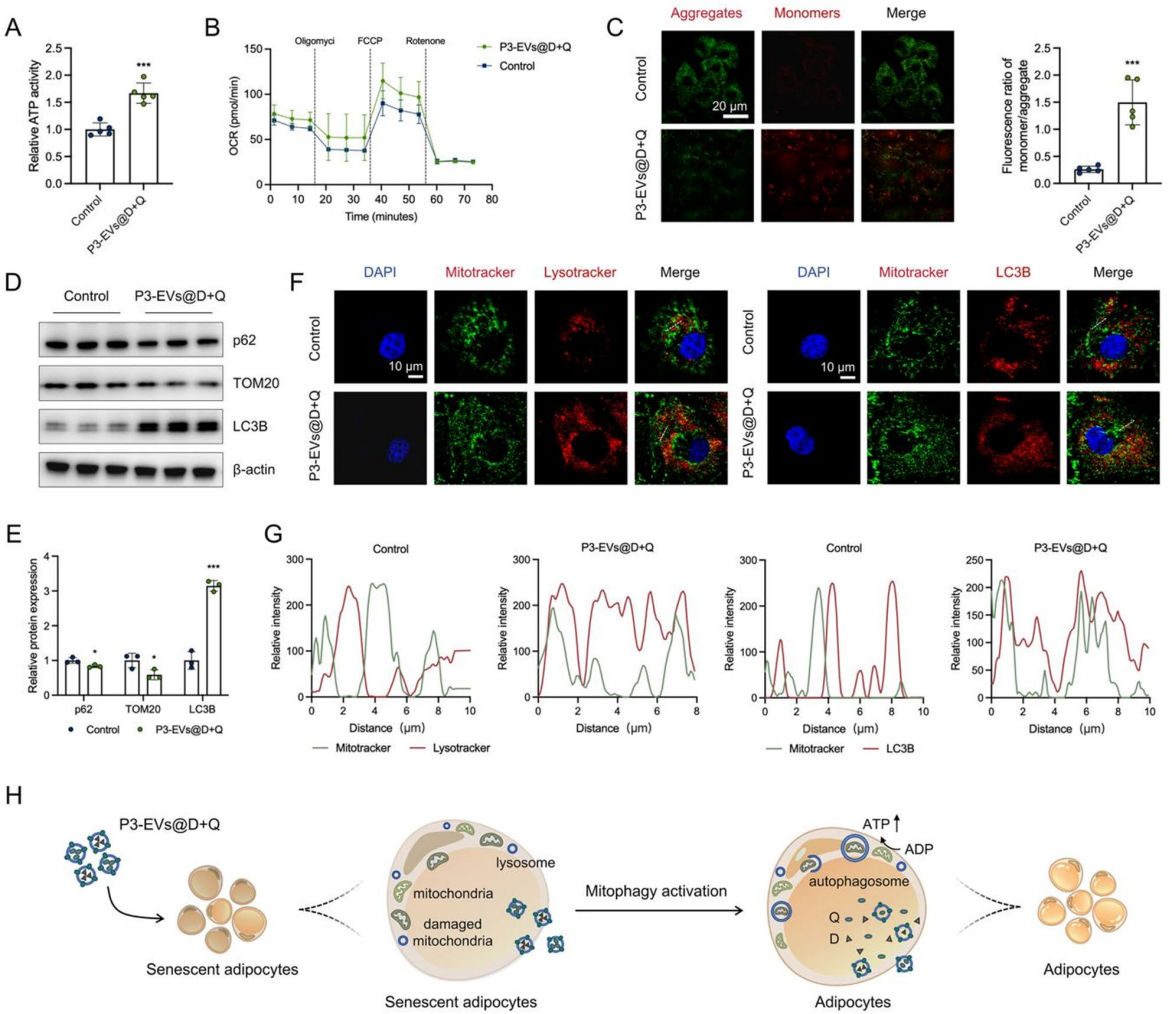
P3-EVs@D + Q promotes mitophagy of senescent adipocytes to maintain mitochondrial homeostasis. (**A**) Relative ATP activity and (**B**) O_2_ consumption rate detection of the two different groups. (**C**) Representative JC-1 staining images and quantification of the monomer/aggregate ratio. (**D**) Western blotting images and (**E**) corresponding quantifications of mitophagy-related proteins, including p62, TOM20 and LC3B. (**F**) Fluorescent visualization of mitophagy localization marked by mitotracker and lysotracker/LC3B and (**G**) intensity profiles across the cell along the selected white dotted line. (**H**) Schematic diagrams of the potential effects of P3-EVs@D + Q on senescent adipocytes. Following the uptake of P3-EVs@D + Q by senescent adipocytes, dasatinib and quercetin were released in a sustained manner, enhancing mitophagy activation and mitochondrial energy metabolism, and ultimately improving cellular function. *P < 0.05; ***P < 0.001; compared to the control group

Considering that mitophagy commonly leads to active mitochondrial activity and ATP synthesis, we first detected the ATP concentration of senescent adipocytes induced by H_2_O_2_ after P3-EVs@D + Q treatment. The results showed that the ATP activity of treated adipocytes was 1.7-fold higher than in the control group (Fig. 9A). The OCR was also significantly increased in the P3-EVs@D + Q group (Fig. 9B), suggesting an increased mitochondrial activity. Moreover, JC-1 staining was performed to evaluate mitochondrial membrane potential. While the control group displayed an intensive green fluorescence, adipocytes treated with P3-EVs@D + Q exhibited predominant red fluorescence with an significantly enhanced red/green intensity ratio (Fig. 9C), indicating the restoration of mitochondrial membrane potential. These parts of results revealed that damaged mitochondrial metabolism in senescent adipocytes was effectively rescued by P3-EVs@D + Q.

Next, a series of mitophagy-related proteins were detected through western blotting. As shown in Fig. 9D-E, the expression levels of p62 (a key adaptor protein involved in mitophagy) and TOM20 (a mitochondrial outer membrane protein) were significantly decreased in the P3-EVs@D + Q group, compared to the control. In contrast, the expression level of LC3B, a classic marker protein involved in mitochondrial autophagosomes formation, was markedly increased after P3-EVs@D + Q treatment (Fig. 9D-E), suggesting the enhanced mitophagy activation. Co-localization fluorescence staining was also conducted using mitotracker and lysotracker to visualize the overlap between mitochondria and lysosomes. After P3-EVs@D + Q administration, a large proportion of mitochondria were observed colocalized with lysosomes (Fig. 9F-G). Similarly, the treatment group also exhibited significantly more co-localization signals of mitotracker and LC3B than the control group (Fig. 9F-G), revealing a promoted autophagosome formation. Taken together, these findings collectively indicated that dasatinib and quercetin released from P3-EVs@D + Q enhanced mitochondrial energy metabolism of senescent adipocytes through activation of mitophagy, which consequently contributed to inhibition of senescence phenotype and improvement of cellular function (Fig. 9H).

## Conclusion

Collectively, this study highlighted the crucial role of adipose tissue as a “hotspot” organ for SASP in the process of systemic aging. To selectively intervene in the senescent phenotype of adipose tissue, we successfully constructed a precise drug delivery system P3-EVs@D + Q. On the basis of edited EVs with stable expression of P3 peptide on membrane surface, we incorporated classic anti-aging drugs dasatinib and quercetin to achieve sustained release. Expectedly, this delivery system enables precise targeting of adipose tissue in vivo and effectively inhibits senescence phenotypes and SASP at both the cellular and tissue levels in vitro. It also remarkably improves the motor performance and basal metabolic capacity of aged mice. Mechanistically, the selectively internalized P3-EVs@D + Q release dasatinib and quercetin, which enhance mitochondrial energy metabolism of senescent adipocytes through activation of mitophagy and consequently suppress the senescent metabolism. Despite the valuable insights provided by this study, there are several limitations. Firstly, the oxidative stress-induced in vitro senescence model of adipocytes was inadequate to entirely replicate the natural aging process in vivo, and additional cellular senescence models should be explored. Secondly, the detailed mechanisms by which dasatinib and quercetin modulates mitophagy in senescent adipocytes, such as potential changes in calcium ion flux, require further comprehensive investigations. Additionally, the time-dependent retention and release dynamics of EV-based drug delivery systems in vivo have yet to be comprehensively defined, and will require the integration of more advanced imaging and labeling approaches in future investigations.

Overall, this study presented a promising EVs-based anti-aging strategy for precisely targeting adipose tissues, inhibiting the senescent metabolism and enhancing the motor performance. Such specific treatment may have broader applications in addressing adipose-related disorders and provide a potential paradigm for organ-specific anti-aging therapies.

## Electronic Supplementary Material

Below is the link to the electronic supplementary material.

Additional file 1.

## Data Availability

All data needed to evaluate the conclusions in the paper are present in the paper and/or the Supplementary Materials.
