# Supplementary material for: Suppression of senescent metabolism of adipose tissue by rebalancing mitochondrial homeostasis via a selective drug delivery system
Source: J Nanobiotechnology. 2026 Jun 2;24:722. doi: 10.1186/s12951-026-04594-w (PMC13445808; doi:10.1186/s12951-026-04594-w)
Supplement: Supplementary file 1 [file 12951_2026_4594_MOESM1_ESM.docx]

**Supplementary Information**

**Suppression of senescent metabolism of adipose tissue by rebalancing mitochondrial homeostasis via a selective drug delivery system**

Liwei Wang^1,2,3,#^, Kaicheng Xu^1,2,3,#^, Ziye Guo^1,2,3,#^, Xiaoyong Wu^1,2,3,#^, Di Wang^1,2,3^, Liang Chen^1,2,3^, Donghua Huang^1,2,3^, Kaile Wu^1,2,3^, Yubin Zhao^1,2,3^, Minjun Yao^1,2,3^, Liming Zheng^1,2,3^, Xiaobo Yang^1,2,3^, Chenyi Ye^1,2,3^, Wushi Cui^1,2,3^, Wanli Li^1,2,3,*^, An Liu^1,2,3,*^, Mao Zhang^4,5,6,*^ and Jianbin Xu^1,2,3,*^

^1^Department of Orthopedic Surgery, the Second Affiliated Hospital, Zhejiang University School of Medicine, Hangzhou 310009, P. R. China

^2^Key Laboratory of Motor System Disease Research and Precision Therapy of Zhejiang Province, Hangzhou 310009, P. R. China

^3^Orthopedics Research Institute of Zhejiang University, Hangzhou 310009, P. R. China

^4^Department of Emergency Medicine, Second Affiliated Hospital of Zhejiang University School of Medicine, Hangzhou 310009, China

^5^Key Laboratory of The Diagnosis and Treatment of Severe Trauma and Burns of Zhejiang Province, Hangzhou 310009, China

^6^Clinical Research Center for Emergency and Critical Care Medicine of Zhejiang Province, Hangzhou 310009, China

**Supplementary Table 1 RT-PCR primers used in this study.**

| **Gene(mouse)** | **Forward (5’ to 3’)** | **Reverse (5’ to 3’)** |
| --- | --- | --- |
| GAPDH | AGGTCGGTGTGAACGGATTTG | TGTAGACCATGTAGTTGAGGTCA |
| P16 | CGCAGGTTCTTGGTCACTGT | TGTTCACGAAAGCCAGAGCG |
| P21 | CCTGGTGATGTCCGACCTG | CCATGAGCGCATCGCAATC |
| MCP1 | TTAAAAACCTGGATCGGAACCA | GCATTAGCTTCAGATTTACGGGT |
| Pai1 | TTCAGCCCTTGCTTGCCTCT | ACACCTTTTACTCCGAAGTCGGT |
| PPARγ | TCGCTGATGCACTGCCTATG | GAGAGGTCCACAGAGCTGATT |
| CEBPα | CAAGAACAGCAACGAGTACCG | GTCACTGGTCAACTCCAGCAC |

**Supplementary Figure 1** Representative *in vivo* fluorescence imaging of epididymal and subcutaneous adipose tissues after treatment with DiO-labelled EVs or P3-EVs via tail intravenous injection in mice (i, ii, and iii denote three independent biological replicates).

**Supplementary Figure 2** Representative *in vivo* fluorescence imaging of heart, liver, spleen, lung, and kidney tissues harvested from mice treated with DiO-EVs or DiO-P3-EVs (i, ii, and iii represent three independent biological replicates).

**Supplementary Figure 3 Adipogenic differentiation of SVF cells.** (**A**) Oil red O staining results after 6 days of adipogenic induction following the standard protocol. The region outlined by the black dashed box is shown at higher magnification on the right. (**B**) Relative mRNA expression levels of adipogenesis markers, including PPARγ and CEBPα. SVF, stromal vascular fraction. ****P* < 0.001.

**Supplementary Figure 4 Indirect calorimetry reveals metabolic alterations in aged mice treated with P3-EVs@D+Q.** Indirect calorimetry analysis was conducted in 18-month-old C57BL/6 mice treated with P3-EVs@D+Q or control (n = 3 per group). (**A**) Respiratory exchange ratio (RER). (**B**) Oxygen consumption (VO₂). (**C**) Energy expenditure (EE). (**D-F**) Locomotor activity, including X-axis beam breaks (D), cumulative distance (E), and velocity (F). (**G**) Body weight measured during the metabolic cage experiment. (**H**) Area under the curve (AUC) of VO₂ calculated over the 24-h period. (**I**) Body weight comparison between groups. Data are presented as the mean ± SD. Statistical analysis was analyzed using two-way ANOVA. *, *P*< 0.05, **, *P*< 0.01, ****P* < 0.001.

**Supplementary Figure 5** The uncropped, original western blot images corresponding to the data presented in Figure 3G.

**Supplementary Figure 6** The uncropped, original western blot images corresponding to the data presented in Figure 4F.

**Supplementary Figure 7** The uncropped, original western blot images corresponding to the data presented in Figure 5C.

**Supplementary Figure 8** The uncropped, original western blot images corresponding to the data presented in Figure 9D.
